# The impact of excessive maternal weight on the risk of neuropsychiatric disorders in offspring—a narrative review of clinical studies

**DOI:** 10.1007/s43440-024-00598-1

**Published:** 2024-04-22

**Authors:** Magdalena Kacperska, Józef Mizera, Maciej Pilecki, Lucyna Pomierny-Chamioło

**Affiliations:** 1 Department of Toxicology, Jagiellonian University Medical College, Medyczna 9, 30-688 Kraków, Poland; 2 Department of Psychiatry, Jagiellonian University Medical College, Kopernika 21a, 31-500 Kraków, Poland

**Keywords:** Maternal body weight, Offspring, Risk of neuropsychiatric disorders, ASD, ADHD, Schizophrenia, Internalizing and externalizing problems

## Abstract

The global prevalence of overweight and obesity is a significant public health concern that also largely affects women of childbearing age. Human epidemiological studies indicate that prenatal exposure to excessive maternal weight or excessive gestational weight gain is linked to various neurodevelopmental disorders in children, including attention deficit hyperactivity disorder, autism spectrum disorder, internalizing and externalizing problems, schizophrenia, and cognitive/intellectual impairment. Considering that inadequate maternal body mass can induce serious disorders in offspring, it is important to increase efforts to prevent such outcomes. In this paper, we review human studies linking excessive maternal weight and the occurrence of mental disorders in children.

## Introduction

Over the last few decades, obesity has become one of the most pressing social and health complications globally. According to the World Health Organization’s Obesity and Overweight report, 1.9 billion people worldwide were overweight in 2016, of which 650 million were obese [1]. The main causes include improper diet, limited physical activity, and a sedentary lifestyle. The problem of excessive body weight also affects pregnant women and young mothers. In 2014, the number of overweight pregnant women was estimated at 38.9 million, including 14.6 million who were obese [2]. Being overweight is classified based on the body mass index (BMI) of 25 to 30, obesity with a BMI between 30 and 35, and severe obesity with a BMI above 35. The Institute of Medicine and National Research Council recommends the range of weight gain during pregnancy depending on the pre-pregnancy BMI.

Owing to extensive research over past years, it has become clear that excessive maternal body weight also has negative health consequences for the next generation. It is considered one of the components of the phenomenon called Developmental Origins of Health and Disease (DOHaD) or fetal programming [3], in which prenatal and early postnatal exposure of the organism to environmental factors, like maternal obesity, unbalanced diet, stress, and infections leading to maternal immune activation, results in permanent changes in children, predisposing them to various diseases in adulthood. Epidemiological studies showed that, for children, maternal obesity before and during pregnancy is a significant risk factor for the development of obesity [4], asthma, stroke [5], hypertension, and insulin resistance [6]. In addition to typically metabolic diseases, maternal obesity has also been linked to behavioural, cognitive, and psychiatric disorders in childhood and adolescence [7].

Maternal obesity has also been reported to impact child eating disorders (food addiction, anorexia, or bulimia), substance addiction [8, 9], intellectual disability or cognitive deficits, sleep problems, aggressive behaviour, and many other psychiatric disorders [9, 10]. The neurobiological processes underlying those abnormalities are diverse [11], thus maternal obesity or other metabolic conditions during pregnancy and gestation could be recognized as a potential, rather than a specific, factor increasing the risk of the mentioned psychiatric disorders in children. Further studies are required to determine whether some disorders are more likely to occur than the others [12].

This review aims to discuss whether the mother’s obesity or being overweight before and during pregnancy has an impact on the neuropsychiatric development of her offspring. We reviewed clinical trials linking excessive maternal body weight with neuropsychiatric disorders in children (autism spectrum disorder (ASD), attention deficit hyperactivity disorder (ADHD), internalizing and externalizing problems, schizophrenia, and cognitive/intellectual impairment). The results of cohort studies, case–control studies, meta-analyses, and systematic reviews were summarized separately. Given the large amount of published data, the present study gives a broad insight into the association between excessive maternal body weight and the development of various neuropsychiatric disorders in children.

## Data sources

The PubMed, Web of Science, and Google Scholar databases were searched to identify the applicable studies. The purpose of this review was to summarize the currently-available data derived from human studies and associate the impact of maternal risk factors, such as obesity, BMI, and Gestational Weight Gain (GWG), on child neurodevelopmental disorders (ASD, ADHD, schizophrenia, anxiety, and depression, etc.) using keywords from 3 principal groups and a search combining groups: (1) ADHD or ASD or schizophrenia or anxiety or depression or neurodevelopmental or internalizing/externalizing problems; (2) pregnancy or offspring or maternal; (3) obesity or overweight or BMI or GWG. Relevant articles, including experimental research, systematic reviews, meta-analyses, and cohort studies published after 2010, were reviewed and synthesized to form the basis of this narrative review, however, for systematic purposes, some older publications have also been included.

## Autism spectrum disorder (ASD)

ASD is one of the neurobiological dysfunctions with an unexplored influence of prenatal environmental factors. ASD is typically diagnosed in early childhood, a lifelong neurodevelopmental disorder characterized by persistent deficits in the ability to initiate and sustain reciprocal social interaction and social communication with the additional presence of several restricted, repetitive, and inflexible patterns of behaviour, interests, or activities [13]. Large cohort studies from Finland [14], USA (California) [15, 16], and Sweden [17] reported a significant association between maternal weight during pregnancy and the risk of ASD in children (Table 1). The results showed that the occurrence of ASD in children of obese mothers was 25–28%, 60%, or even 94% higher, respectively, compared to children of mothers with a normal BMI. The Kong et al. (2018) cohort study revealed that pre-pregnancy (PP) maternal obesity (MOb) was associated with a slightly increased risk of psychiatric disorders and mild risk of neurodevelopmental disorders in children. These risks were significantly higher in the offspring of severely obese mothers and when comorbid with gestational or pre-gestational diabetes [14]. The follow-up time in this study was not the same for the entire cohort, so this data may be underestimated. BMI was examined only at 1 point, therefore GWG was not assessed. Maternal PP BMI > 30 was reported to be associated with a 37% higher likelihood of ASD and gastrointestinal disturbances comorbidity in children [16]. The authors point out the importance of maternal immune activation as a factor predisposing the occurrence of ASD in children. Excessive adipose tissue triggering immune activation in pregnant women is a suggested mechanism involved in the impairment of brain maturation in children [16, 18, 19]. In a multisite case–control study in the USA, severe obesity (BMI > 35) in mothers led to an almost 87% higher risk of ASD [20], while the prospective birth cohort study showed that each 5-point increase in maternal PP BMI was related to a 38% increase in ASD risk occurrence in children [21]. This study used parents’ reports regarding ASD diagnosed by a doctor based on special questionnaires, but the consistency of the information with medical records was not confirmed. Although Matias et al. (2021) did not find a significant association between pregnancy maternal overweight (PP MOv) and ASD risk, the meta-analysis, which included 36 cohorts, demonstrated that the children of overweight mothers are 10% more likely to be diagnosed with ASD [22]. Furthermore, when a mother’s BMI was higher than 30, the risk of ASD occurrence increased to 36%. Moreover, a large retrospective cohort study in Sweden showed that increasing the mother’s pre-pregnancy BMI value above 21 was associated with a higher risk of ASD. Surprisingly, the results from the fathers’ BMI analysis and the sibling analysis suggest that these results are influenced by residual confounding factors and that the mothers’ BMI may be a proxy for other risk factors shared by members of the same family, such as genetics [17].

**Table 1:** The risk of developing ASD in child due to obesity or overweight of mother before or during pregnancy in epidemiological human studies

Offspring risk of outcome: autism Spectrum Disorder (ASD)			
Maternal factor	References	Sample	Results in children
Cohort studies			
⬆ PP BMI	[14]	649,043	↑ risk: 28%
	[17]	333,057	↑ risk: 94% (for both maternal and paternal obesity; attenuated to non-significance in matched-siblings analysis)
	[21]	1164	positively associated with an increased risk of ASD
			↑ risk: 38% (for each 5-points increase in BMI)
	[16]	308,536	↑ risk: 25%
⬆ GWG	[26]	129,733	Significantly associated with an increased risk of ASD
			(no association between insufficient GWG and ASD risk)
	[17]	333,057	Significantly associated with an increased risk of ASD
			(also in matched-siblings analysis)
Case–control studies			
MOb	[15]	1004	↑ risk: 60%
⬆ PP BMI	[20]	4409	Severe obesity (BMI > 35): ↑ risk: 154%
			(87% when adjusted for confounders)
⬆ GWG	[23]	2941	Association between excessive GWG and ASD risk
	[24]	10,920	Significantly associated with an increased risk of ASD
			(risk increases for every 5 pounds of weight gained)
	[25]	776	Association between excessive GWG and ASD risk
			↑ ASD risk: 6% (for each 5-pound increase of GWG)
	[20]	4409	↑ risk: 29% (22% when adjusted for confounders)
Systematic review			
⬆ GWG	[66]	6 cohort	Association between excessive GWG and ASD risk (no association between insufficient GWG and ASD risk)
		3 case–control	
Meta-analysis			
PP MOb	[22]	36 cohort studies	↑ risk: 36%
	[67]	15 cohort studies	↑ risk: 37%
MOv	[22]	36 cohort studies	↑ risk: 10%
	[67]	13 cohort studies	↑ risk: 9%
⬆ GWG	[27]	5 cohort studies	↑ risk: 10% (cohort studies)
		4 case–control	↑ risk: 38% (case–control studies)

*PP* pre–pregnancy, *BMI* Body Mass Index, *ASD* Autism Spectrum Disorder, *MOb* Maternal Obesity, *GWG* Gestational Weight Gain, *Mov* Maternal Overweight

Independently, GWG is also considered a critical factor for a higher ASD risk. Three cohort studies suggest that its role is even more crucial than that of PP MOb [17, 23, 24]. The data also showed that, even in matched-siblings analysis, the association with GWG is still present while the link with PP MOb is attenuated [17]. Other studies indicate that the association between GWG and ASD in children is further strengthened by MOv/MOb [23, 25]. In turn, research by Dodds et al. (2011) indicates that, in genetically ASD-susceptible children, factors such as high maternal BMI or GWG play a minor role [26]. In another study, the exact association between GWG and the risk of ASD was estimated: each 5-pound (~ 2.26 kg) rise in GWG increased the odds of ASD by 10–17% [24]. Windham et al. (2019) also obtained comparable results, where the risk of ASD was estimated at 6% for each 5-pound increase in GWG [25]. Interestingly, an elevated risk of ASD was observed for excessively low GWG, providing evidence that maternal undernutrition during pregnancy may also contribute to ASD risk [17].

The link between GWG and ASD risk in children is supported by meta-analysis (based on 5 cohort studies and 4 case–control studies), which showed that excessive GWG increased ASD risk by 10% (for cohort studies) and 38% (for case–control studies) [27]. However, this meta-analysis did not confirm the significant correlation between the risk of ASD in children and inadequate maternal GWG (including both higher and lower rates than recommended).

Summarizing, numerous studies confirm a positive correlation between high BMI before pregnancy and the occurrence of ASD in children, however, excessive GWG seems to be a more critical factor, additionally reinforced by MOv and MOb. The literature reports a significant impact of other factors predisposing the development of ASD in children, such as pre-eclampsia, pre-gestational diabetes, gestational diabetes [17], cholesterol level, or dyslipidemia [14]. However, it should be noted that elevated BMI is a core feature defining the metabolic syndrome and is strongly associated with the development of the metabolic pregnancy complications listed above.

## Attention deficit hyperactivity disorder (ADHD)

ADHD is a neurodevelopmental disorder typically diagnosed in childhood, and its features may also be observed in adulthood. Axial symptoms of ADHD include inattention and/or hyperactivity-impulsivity, which has a direct negative impact on the academic/occupational or social functioning of a person [28].

The exact aetiology of ADHD remains unclear. Research results indicate complex determinants of ADHD, including genetic predispositions and the influence of environmental factors. The data on the influence of the mother’s BMI and GWG on the risk of ADHD symptoms in the child is not as clear as in the case of ASD. Recent studies suggest that environmental factors, such as PP and pregnancy MOv and/or MOb, contribute to an increased risk of ADHD in children (Table 2). Analysis of the results of the first large prospective cohort study showed that children of women who were overweight and gained significant weight during pregnancy were twice as likely to develop ADHD symptoms compared to women with normal body weight [29]. These results persisted after adjustment for various confounding factors, such as smoking during pregnancy, gestational age, weight gain, birth weight, infant sex, mother’s age, mother’s education, and family structure. There was also an increased risk of ADHD symptoms among the children of slim mothers who lost weight, suggesting that complications rather than weight are an important factor. It should be noted that, in this study, the assessment of the presence of ADHD symptoms was made by teachers working with children, which was not synonymous with diagnosis. All symptoms were significantly more common in boys than in girls, consistent with the known sex-dependent distribution of ADHD [29]. Subsequent cohort studies confirmed that PP MOv and PP MOb (but not underweight) were dose-dependently associated with an increased risk of ADHD in children [30–32]. However, after taking into account comparisons with siblings, these relationships were weakened. Therefore, the authors attributed the relationship between overweight/obesity in the mother before pregnancy and ADHD in children to unmeasured family complications, e.g., genetics [31]. Although increased PP maternal BMI was shown to be a significant factor, no significant relationship for GWG was found [29, 30, 33]. The results of recent research have shown that the risk of ADHD is dependent on the trimester of gestation in which excessive weight gain occurs. Thus, they indicated that slow weight gain in the second trimester followed by rapid weight gain in the third trimester most significantly increases this risk [34]. According to the results of two conducted independent meta-analyses, the risk of ADHD occurrence in the children of overweight mothers is 30% higher compared to those of normal weight [22, 35]. MOb, in turn, increased the risk of ADHD by up to 62% [22], or 59% after adjustment for known risk factors, such as smoking, maternal age, birth weight, prematurity, and Caesarean section, in the long-term prospective study (17.7 years) by Perea et al. (2022). This risk increased to 66% with a diagnosis of gestational diabetes [33], and when the combined contributions of maternal weight and excessive weight gain (EWG) were assessed, these two factors together were associated with the highest risk of ADHD (2.13 times the risk when compared to normal weight without EWG). When the influence of measured confounding factors (i.e., maternal age, maternal education, smoking, year and country of birth, and sex of the child, etc.) was taken into consideration, the data showed a lower impact of obesity and being overweight on the risk of ADHD occurrence (21 and 60%, respectively). Li et al. (2021) suggested that the impact of unmeasured familial confounding factors could further attenuate the link between MOb and ADHD [36], while a recent cohort study showed that PP MOb, but not MOv, is associated with a higher risk of ADHD symptoms [37].

**Table 2:** The risk of developing ADHD in child due to obesity or overweight of mother before or during pregnancy in epidemiological human studies

Offspring risk of outcome: attention deficit hyperactivity disorder (ADHD)			
Maternal factor	References	Sample	Results in children
Cohort studies			
⬆ PP BMI	[29]	3 cohorts	MOb associated with teacher-rated ADHD symptoms
		(12,566 children)	
	[48]	2634 children	MOb associated with teacher-rated ADHD symptoms
			(but not with mother-rated)
	[31]	673,632	MOb associated with an increased risk of offspring ADHD. Association not present in sibling comparisons
	[30]	174	MOb associated with an increased risk of ADHD symptoms
	[32]	843	MOb, but not overweight associated with increased ADHD symptoms at 4 years of age
	[37]	1428	MOb, but not overweight associated with an increased risk of ADHD symptoms
	[33]	1036	59% higher risk of ADHD, adjusted for known risk factors, for the offspring of women with MOb
⬆ GWG	[34]	57,822	↑ ADHD risk: 31%
Meta-analysis			
PP MOb	[22]	36 cohort studies	↑ ADHD risk: 62%
	[36]	8 cohort studies	↑ risk: 92%
			(60% or less when adjusted for confounders)
	[35]	10 cohort studies	↑ risk: 65%
	[67]	10 cohort studies	↑ risk: 55%
MOv	[22]	36 cohort studies	↑ risk: 30%
	[36]	8 cohort studies	↑ risk: 30%
			(21% or less when adjusted for confounders)
	[35]	10 cohort studies	↑ risk: 30%
	[67]	10 cohort studies	↑ risk: 21%
Umbrella review			
Obesity	[38]	63 meta-analyses	Evidence of association: convincing (class I)
Overweight			Evidence of association: highly suggestive (class II)

*PP* pre–pregnancy, *BMI* Body Mass Index, *ASD* Autism Spectrum Disorder, *MOb* Maternal Obesity, *GWG* Gestational Weight Gain, *Mov* Maternal Overweight

An umbrella review also confirmed the positive correlation between MOb and ADHD in children. The authors define MOb as a convincing factor for the higher risk of ADHD occurrence in children and place it amongst four other factors most associated with the risk of ADHD (i.e., childhood eczema, hypertensive disorders during pregnancy, preeclampsia, and exposure to acetaminophen during pregnancy). They suggest that the impact of MOb may be more significant than maternal smoking during pregnancy. Although these associations remain robust, the authors note that other confounding factors should be taken into consideration [38].

The above data indicates a strong relationship between PP MOb and ADHD symptoms in children. Moreover, numerous studies indicate that MOv may also contribute to the development of this disorder. It seems to be valuable to assess this relationship in the context of the child’s sex. Frazier et al. (2023) found that both MOv and MOb affect child development differently depending on sex, but research specifically on ADHD in this area is very limited [7].

## Schizophrenia

Schizophrenia is a severe mental disorder characterized by the presence of several disturbances in the areas of thinking, perception and self-perception, cognition, volition, affect, and behaviour [13]. It is most often diagnosed in early adulthood and, despite treatment progress, it is a significant cause of disability. Only a limited number of studies have examined the impact of MOb on the higher risk of schizophrenia in children (Table 3). Existing data shows an increased risk of schizophrenia in children for excessive, but also deficient, maternal BMI rates.

**Table 3:** The risk of developing schizophrenia in child due to obesity or overweight of mother before or during pregnancy in epidemiological human studies

Offspring risk of outcome: schizophrenia			
Maternal factor	References	Sample	Results in children
Cohort studies			
PP BMI (obesity)	[39]	11,017	↑ risk: twofold (BMI > 29)
			(not significant when adjusted for confounders)
	[41]	6633	↑ risk: threefold (BMI > 30)
			(significant when adjusted for confounders)
Case–control studies			
⬆ GWG	[42]	336	↑ risk: 24% for every one-unit BMI (early pregnancy)
			↑ risk: 19% for every one-unit BMI (late pregnancy)
Systematic review			
⬆ BMI	[40]	4 cohort studies	Association between PP BMI (over 29 or 30)
			↑ risk: two- to three-fold

*PP* pre – pregnancy, *BMI* Body Mass Index, *GWG* Gestational Weight Gain

A 28-year follow-up study conducted on the Finnish birth cohort suggests a two-fold increased risk of schizophrenia for children of obese mothers (BMI > 29) compared to those of mothers with a BMI of 19.1–29.0 [39, 40]. However, when adjusted for confounders (sex, social class, age of mother), these findings were no longer statistically significant [39]. A study conducted on the American cohort born between 1959 and 1967 reports an almost three-fold increased risk of schizophrenia and spectrum disorders due to maternal PP BMI > 30 compared to the average BMI (20.0–26.9). Unlike the Finnish experiment, these findings were significant even after adjusting for confounders, such as maternal age, parity, race, education, and cigarette smoking during pregnancy, but no such relationship was found when the mother’s BMI was lower than 19.9 [41]. Furthermore, Kawai et al. (2004) investigated the relationship between the risk of schizophrenia in children and BMI, including BMI during early and late pregnancy [42]. They found a significant association between the increased risk of schizophrenia for each unit of maternal BMI in both early (24% higher risk) and late (19% higher risk) pregnancy. It should also be mentioned that a low mother’s BMI may predispose the development of schizophrenia in the child.

On the other hand, the study based on cohort births from 1924 to 1933 in Finland reported that the risk of schizophrenia in children was related to low maternal BMI (< 24) rather than to BMI > 30. The authors consider their findings to be different from more recent observations due to the dissimilar economic situation in the 1930s. They link their observations with the lack of welfare structures, illness, and malnutrition at that time. These results are also confirmed by observations from a cohort of children in China, conceived during the 1959–1961 famine, which showed a twofold increased risk of schizophrenia [43]. Currently, in Western populations, where nutritional status is good, a higher maternal BMI is more likely to be associated with neurodevelopmental disorders than malnutrition [44].

## Internalizing and externalizing problems

The terms internalizing and externalizing problems are an alternative to the classic diagnostic criteria way of grouping mental disorders and emotional problems that appear in childhood and adolescence. Internalizing problems usually include depression, anxiety, and social withdrawal. Externalizing problems include aggression, lack of cooperation with adults, violations of rules, and impulsivity. These descriptions are supplemented by nosological criteria including major depressive disorder, oppositional defiant disorder, ADHD, and conduct disorders. Together, in a broader sense, they constitute two groups of disorders with certain common features, but having different severity and clinical pictures, often including symptoms from both groups. However, in research on mental disorders of children and adolescents, these terms are often used in both a broader and narrower sense [45].

Epidemiological studies show that internalization and externalization problems diagnosed in children could also be linked to maternal BMI (Table 4). The cohort study on an Australian sample of 2785 offspring-mother pairs has examined internalizing (anxious/depressed) and externalizing (behavioural) problems. During the study, mothers were asked to fill in the Child Behaviour Checklist (CBCL) when their children were 5, 8, 10, 14, and 17 years old. The results confirmed that increased maternal BMI was associated with both internalizing and externalizing problems. Although the intensity of both types of problems diminished with age (as it should have done), in children of mothers with high BMI, a less rapid decrease in anxious/depressed symptoms was reported. These findings were statistically significant even after adjusting for confounders [46]. Robinson et al. (2013), using the same data (same cohort and method), estimated that the risk of affective problems (including depression) for children of obese and overweight mothers was 72 and 51% higher, respectively, compared to the children of mothers with normal PP BMI [47]. Those findings are consistent with the cohort study conducted on the Finnish population of children born between 2004 and 2014, which reported that severely obese mothers (BMI > 35) had a 67% higher risk of having a child with psychosis, mood, and anxiety disorders, compared to children of mothers with normal weight [14]. Similarly, anxiety-related problems have been observed when a mother’s PP BMI was higher than 40 (very severe obesity), but no consistent impact on internalizing problems in children has been revealed [10]. MOb has also been associated with increased emotional dysregulation (sadness and fear) [48], which can be a risk factor for future psychological difficulties, including anxiety and depression [49].

**Table 4:** The risk of developing internalizing and externalizing problems in child due to obesity or overweight of mother before or during pregnancy in epidemiological human studies

Offspring risk of outcome: internalizing and externalizing problems			
Maternal factor	References	Sample	Results in children
Cohort studies			
PP MOb	[46]	2868 children	Association between maternal BMI and higher internalizing (anxious/depressed) problems (significant when adjusted for confounders)
	[47]	2868 children	Dysthymic disorder, major depressive disorder
			↑ risk: 72%
			(significant when adjusted for confounders)
	[50]	2127 white children	4.3-unit increase in the BPI total scores and ↑ risk: 78% (similar in externalizing and internalizing subscales)
		1268 African American children	no significant relationship
	[10]	112 children	Association between maternal BMI and higher externalizing, anxious/depressed scale scores (data based on CBCL)
			(very severe obesity: BMI > 40)
	[48]	1714	↑ risk: twofold
			(OR = 2.29 according to kindergarten teachers reports)
			(OR = 1.84 according to mothers’ reports)
PP MOv	[47]	2868 children	↑ risk: 51%
			(significant when adjusted for confounders)

*PP* pre–pregnancy, *BMI* Body Mass Index, *MOb* Maternal Obesity, *OR* Odds ratio, *Mov* Maternal Overweight, *BPI* Behavior Problems Index, *CBCL* Child Behavior Checklist

Very interesting and unexpected results were provided by a cohort study described by Tanda & Salsberry (2014) which compared the effect of BMI of White and African American women on the development of internalizing and externalizing disorders in children [50]. PP MOb in White women was associated with an average increase in BPI total score of 4.3 units in the Behavioral Problems Index (BPI) compared with mothers who were of normal weight before pregnancy, with other factors held constant. PP MOv was marginally associated with BPI total scores, holding other factors constant (p = 0.062). Moreover, maternal underweight was also associated with a greater risk of behavioural problems in children, however, after taking into account maternal age and prenatal smoking, it reduced the size and strength of the association to a non-significant level. On the other hand, among African American children, not only was PP MOb a non-significant contributor to the BPI total score, but the direction of the coefficient was also opposite to that among Whites. Neither PP MOv nor being underweight was associated with children’s total BPI scores. Most of the data came from mothers’ self-reports, including the mother’s pre-pregnancy weight, so weight may be underestimated in obese and overestimated in underweight individuals [50]. The lack of consistency in the association between a child’s behavioural problem scores and maternal pre-pregnancy obesity in both racial groups sheds new light on risk factors. These results indicate that the association between pre-pregnancy maternal obesity and child behaviour problems may be largely driven by unfavourable social characteristics.

## Cognitive impairment and intellectual disability

Data from studies examining intellectual abilities in children further confirms the detrimental impact of excessive maternal BMI, however, there is not full agreement on this matter (Table 5). Most cohort and observational studies connect MOb with the decrease in child IQ points [51, 52] and poor cognitive performance, including motor, spatial, and verbal skills [53, 54]. In a study conducted by Pugh et al. (2015), lower IQ and worse executive function test results were reported in children of obese mothers, however, excessive GWG does not affect the child’s IQ [55]. In turn, another study reported that there are no solid associations between maternal BMI and cognition in children [56]. In addition, U-shaped associations have been determined, suggesting that inadequately low maternal PP weight is significantly associated with lower IQ rates in children [57–59].

**Table 5:** The risk of developing cognitive impairments in child due to obesity or overweight of mother before or during pregnancy in epidemiological human studies

Offspring risk of outcome: cognitive impairment			
Maternal factor	References	Sample	Results in children
Cohort studies			
⬆ PP BMI	[52]	1783	Reduction in IQ of − 0.40 points for each unit in BMI
			(after adjustment for social factors: − 0.27 point)
	[53]	11,025 (age 5)	A significant association between BMI and cognitive performance
		9882 (age 7)	
	[54]	1967 (Spain)	A significant association between MOb and reduced cognitive development
		412 (Greece)	
			− 2.72 score reduction (Spain)
			− 3.71 score reduction (Greece)
	[57]	6850	Risk of delayed mental development:
			↑ risk: 36% (children of underweight mothers)
			↑ risk: 38% (children of class II and III obese mothers)
	[58]	3212	BMI > 30:
			↓ in IQ: 2.0 point – Full Scale
			↓ in IQ: 2.5 point – Verbal Scale IQ
			Excessive GWG:
			↓ in IQ: 6.5 point
	[59]	355	MOb:
			↓ in general IQ and nonverbal scores of 5.0 points
	[55]	763 mother–child pairs	MOb:
			↓ in IQ: 3.2 point
			↑ 12.7 s needed in executive function test
	[51]	4324 mother-father-child triads	MOb:
			↓ in IQ: 3.4 point
⬆ GWG	[58]	3212	GWG + MOb:
			↓ in IQ: 6.5 point
	[55]	763 mother–child pairs	↑ 15 s needed in executive function test
Meta-analysis			
PP MOb	[67]	7 cohort studies	↑ risk: 40%
PP MOv	[67]	7 cohort studies	↑ risk: 13%

*PP* pre–pregnancy, *BMI* Body Mass Index, *MOb* Maternal Obesity, *GWG* Gestational Weight Gain, *MOv* Maternal Overweight, *IQ* intelligence quotient

## Summary

The number of papers investigating the issue of inadequate maternal BMI and its consequences in children clearly indicates that this topic has gained interest. The issue of parental obesity may be of even greater public health concern as there are animal studies [12, 60] and epidemiological studies [61] reporting multi-generational adverse effects of excess body weight. Most of the data confirms significant adverse neurodevelopmental outcomes in children caused by MOv/MOb and/or excessive GWG. However, epidemiological studies do not provide explanations of the mechanisms involved in the development of the observed disorders. Certainly, all these diseases have multifactorial aetiology and heterogeneous presentation, and the factors discussed in this article are just some of many. Nevertheless, the acquired observations serve as guidance in planning animal studies, which in turn aim at understanding those mechanisms.

The mother’s weight may influence the child’s mental condition through various mechanisms. The first and most obvious of these may be related to the common genetic risk of obesity and mental disorders. Co-occurring obesity and mental disorders in mothers may also affect the quality of nutrition, bonding with the child, and the child’s emotional self-regulation. The occurrence of obesity is also associated with the presence of several other risk factors for mental disorders, such as physical activity level, alcohol consumption, and socio-economic status [62]. Each of the above-mentioned individual risk factors may have consequences in the occurrence of further phenomena increasing the risk of mental disorders [63]. Maternal obesity may affect the course of pregnancy itself—the mother’s health during pregnancy, the length of pregnancy, and the birth weight of the child [64]. A relatively large amount of clinical data refers to the epigenetic impact of maternal obesity on the child’s development, including the development of the central nervous system, through the negative consequences of hypothalamic–pituitary–adrenal (HPA) axis deregulation, early-onset intensification of inflammatory processes, and oxidative stress. The transition of microbiota between the mother and the child may also be important. All these phenomena can co-occur and influence each other [64, 65].

The research has many limitations. The most important of them is related to the use of different diagnostic criteria for disorders and diagnostic strategies in different studies. This especially limits conclusions regarding anxiety and depressive disorders, which were based on both clinical diagnoses and self-report tools or descriptions of the child’s behaviour. Moreover, paternal data and parental genetic information were unavailable in the study, so paternal factors and genetic contributions to the likelihood of disease were not controlled. There may be other potential confounding factors, such as environmental exposures and postnatal risk factors, like childhood diet and household exposures, that could not be controlled in the studies. Research on maternal obesity and its impact on the risk of mental disorders should also differentiate the nature of maternal obesity and the dynamics of its increase. Did obesity occur in adolescence or did it appear later in life? How much weight was gained during pregnancy? Is obesity associated with the failure to follow the rules of a healthy diet? These are some of the questions that would allow for a more thorough examination of the analysed relationships.

Since there are no effective measures to counteract harmful effects in children once they have occurred, prevention appears to be the best strategy. Body weight is controlled during pregnancy, and its gain is a potentially modifiable factor. It seems very important to control proper weight gain, taking into account PP BMI, by the recommendations of the Institute of Medicine and the National Research Council, and likewise aim to maintain a healthy BMI when planning a pregnancy. The above data should help implement early interventions for mothers of high-risk infants.

## Data Availability

Not applicable.

## Abbreviations

ADHD: Attention deficit hyperactivity disorder
ASD: Autism spectrum disorder
BMI: Body mass index
DOHaD: Developmental origins of health and disease
EWG: Excessive weight gain
GWG: Gestational weight gain
PP: Pre-pregnancy
MOv: Maternal overweight
MOb: Maternal obesity
